# Longitudinal study assessing the return of chloroquine susceptibility of *Plasmodium falciparum* in isolates from travellers returning from West and Central Africa, 2000–2011

**DOI:** 10.1186/1475-2875-12-35

**Published:** 2013-01-25

**Authors:** Myriam Gharbi, Jennifer A Flegg, Véronique Hubert, Eric Kendjo, Jessica E Metcalf, Lionel Bertaux, Philippe J Guérin, Jacques Le Bras

**Affiliations:** 1 Mère et enfant face aux infections tropicales, IRD unité mixte de recherche 216, Université Paris Descartes-Paris V, 4 avenue de l'Observatoire, Paris Cedex 06 75270, France; 2 PRES Sorbonne Paris Cité, Faculté de Pharmacie, Paris, France; 3 WorldWide Antimalarial Resistance Network (WWARN), Paris, France; 4 EHESP Rennes, Sorbonne, Paris Cité, France; 5 WWARN, Oxford, UK; 6 Centre for Tropical Medicine, Nuffield Department of Clinical Medicine, CCVTM, University of Oxford, Oxford, UK; 7 Centre National de Référence du Paludisme, Paris, France; 8 Service de Parasitologie Mycologie, CHU Bichat-Claude Bernard, APHP, Paris, France; 9 Service de Parasitologie Mycologie, CHU Pitié-Salpétrière, APHP, Paris, France; 10 Epidemiology and Infectious Diseases, Department of Zoology, University of Oxford, Oxford, UK; 11 Centre National de Référence du Paludisme, Marseille, France; 12 Unité de Recherche en Physiologie et Pharmacocinétique Parasitaires - UMR-MD3 Relations Hôte-Parasites - Pharmacologie et Thérapeutique, Institut de Recherche Biomédicale des Armées, Marseille, France; 13 UMR S 707: Epidemiology, Information Systems, Modelling, INSERM and Université Pierre et Marie-Curie-Paris, Paris, France

**Keywords:** Plasmodium falciparum, Malaria, Travellers, Chloroquine, Resistance, pfcrt76, *In vitro*, West Africa, Central Africa

## Abstract

**Background:**

Chloroquine (CQ) was the main malaria therapy worldwide from the 1940s until the 1990s. Following the emergence of CQ-resistant *Plasmodium falciparum*, most African countries discontinued the use of CQ, and now promote artemisinin-based combination therapy as the first-line treatment. This change was generally initiated during the last decade in West and Central Africa. The aim of this study is to describe the changes in CQ susceptibility in this African region, using travellers returning from this region as a sentinel system.

**Methods:**

The study was conducted by the Malaria National Reference Centre, France. The database collated the *pfcrtK76T* molecular marker for CQ susceptibility and the *in vitro* response to CQ of parasites from travellers’ isolates returning from Senegal, Mali, Ivory Coast or Cameroon. As a proxy of drug pressure, data regarding CQ intake in febrile children were collated for the study period. Logistic regression models were used to detect trends in the proportions of CQ resistant isolates.

**Results:**

A total of 2874 parasite isolates were genotyped between 2000–2011. The prevalence of the *pfcrt76T* mutant genotype significantly decreased for Senegal (from 78% to 47%), Ivory Coast (from 63% to 37%), Cameroon (from 90% to 59%) and remained stable for Mali. The geometric mean of the 50% inhibitory concentration (IC_50_) of CQ *in vitro* susceptibility and the proportion of resistant isolates (defining resistance as an IC50 value > 100 nM) significantly decreased for Senegal (from 86 nM (59%) to 39 nM (25%)), Mali (from 84 nM (50%) to 51 nM (31%)), Ivory Coast (from 75 nM (59%) to 29 nM (16%)) and Cameroon (from 181 nM (75%) to 51 nM (37%)). Both analyses (molecular and *in vitro* susceptibility) were performed for the 2004–2011 period, after the four countries had officially discontinued CQ and showed an accelerated decline of the resistant isolates for the four countries. Meanwhile, CQ use among children significantly deceased in this region (fixed effects slope = −0.3, p < 10^-3^).

**Conclusions:**

An increase in CQ susceptibility following official withdrawal of the drug was observed in travellers returning from West and Central African countries. The same trends were observed for molecular and *in vitro* analysis between 2004-2011and they correlated to the decrease of the drug pressure.

## Background

Chloroquine (CQ) was the main malaria therapy worldwide between the 1940s and the 1990s, due to its effectiveness, safety, low cost and antipyretic properties. Resistance to CQ emerged in different locations in the late 1950s, first in Southeast Asia (Thai-Cambodian border) and in South America (Colombia and Venezuela) [1-3]. Resistance spread relatively rapidly and was detected in East Africa (Kenya and Tanzania) in the late 1970s, probably as a consequence of an importation of resistant strains from Asia [4,5]. It was first reported in West Africa in the early 1980s [6-8]. Despite evidence of a relatively high prevalence of CQ resistance in Africa for more than two decades, this single drug remained the first-line treatment of uncomplicated *Plasmodium falciparum* malaria recommended in most sub-Saharan African countries until the early 2000s. Consequently, a significant increase of clinical malaria morbidity and mortality in children under five years was attributable to CQ resistance from the 1980s to the 1990s [9-11]. Malawi was the first African country to change its national drug policies from CQ to sulphadoxine-pyrimethamine (SP) in 1993. In time, all malaria-endemic countries on the African continent discontinued the routine use of CQ against *P. falciparum.* The change of policy to artemisinin-based combination therapy (ACT) as first-line treatment for uncomplicated *P. falciparum* malaria occurred in all endemic countries between 2000 and 2009.

The frequency of *pfcrtK76T* mutation in *P. falciparum* has been associated with clinical CQ resistance and represents a good indicator of the parasite’s intrinsic resistance to CQ [12,13]. Since the withdrawal of CQ, previous studies have documented a decrease in the prevalence of CQ-resistant parasites. In East Africa, a decrease has been well described in Malawi after the CQ ban in 1993 and in Kenya after the CQ ban in 1999 [14-16]. A clinical trial conducted in Malawi in 2005 even confirmed the return of *in vivo* CQ efficacy to 99% *versus* less than 50% before 1993 [17]. A few studies in West Africa, particularly in Senegal, have described the same trend for CQ susceptibility after the drug was withdrawn from first-line in 2003 [18,19]. The relationship between drug pressure and trends in CQ susceptibility has been confirmed in several countries where information was available [20].

The assessment of parasites imported from malaria endemic regions is also a potential tool for monitoring malarial drug resistance; that approach has been tested in this study. It is assumed that travellers returning to non-endemic areas with malaria are infected with a wide variety of *Plasmodium* strains which partly reflect the parasite populations in the visited regions. The fact that travellers are likely to be non-immune with a low risk of re-infection also facilitates the detection of truly resistant isolates. This study describes the longitudinal changes in molecular and *in vitro* correlates of CQ resistance in parasites from travellers to West and Central Africa, following the withdrawal of CQ as the recommended treatment.

## Methods

### Data and samples collection

The study was conducted by the National Reference Centre for Malaria (CNR), France in collaboration with the WorldWide Antimalarial Resistance Network (WWARN).

Travellers who returned to France with symptomatic *P. falciparum* infections were included in the study. Four countries, Senegal, Mali, Ivory Coast and Cameroon, had sufficiently large numbers of returnees for meaningful comparison. Data sets from 2000 to 2011, for these countries under consideration, were included in the study. Cases originated in one of 80 hospitals participating in the French sentinel network for malaria. An imported malaria infection was defined by two factors, positive thin and thick blood smear and a recent travel history to one of the four chosen countries in the two months prior to diagnosis, without evidence of autochthonous or transfusion-related transmission. Each case was prospectively registered in the French national database after medical records were checked. Basic demographic and epidemiologic data, clinical and parasitological information, treatment, history of travel and malaria infection were collected systematically. Blood samples were collected from about half of the French hospital network, which document anti-malarial drug resistance, for molecular and *in vitro* analyses. Only samples with parasitaemia above 0.1% were analysed *in vitro*. No informed consent was required for this study as all following procedures are part of the routine French national surveillance system of malaria.

### Molecular analysis

A total of 2,874 pre-treatment isolates were collected between 2000–2011. Before 2006, all the samples were systematically and prospectively analysed by the PCR-RFLP method to distinguish the *pfcrt76K* from the *pfcrt76T* allele related to CQ resistance [19,21]. After 2006, because of financial constraints, only 30 samples per year and per country were randomly selected for a retrospective molecular analysis. DNA was extracted from blood samples for molecular analysis using the QIAamp DNA Mini Kit, Qiagen® before 2008 and the MagNA Pure LC DNA Isolation Kit I, Roche after 2008.

### *In vitro* assay

A total of 1,483 fresh venous blood isolates taken before treatment were centralized and tested for *in vitro* susceptibility between 2000–2011. Thin blood smears were examined to determine *P. falciparum* density and *P. falciparum* mono-infection. The batches of plates were tested and validated on the CQ-susceptible 3D7 reference strain (Africa) and the CQ-resistant W2 reference strain (Indochina) using the standard 42-hour ^3^H-hypoxanthine uptake inhibition method in controlled atmospheric conditions in the incubator (5% CO_2_, 10% O_2_ and 85% N_2_) [22-24]. *In vitro* isotopic microtests were performed, aliquoting 200 μl/well of the suspension of parasitized erythrocytes into 96-well plates pre-dosed with anti-malarial drugs. Radioactivity incorporated by the parasites was measured using a scintillation counter. The *in vitro* susceptibility was determined by measuring the concentration of drug required to inhibit parasite growth by 50% (50% inhibitory concentration (IC_50_)) for each of the isolates [25,26]. The IC_50_ value was calculated using the inhibitory sigmoid Emax model, with estimation of the IC_50_ through nonlinear regression [27]. Susceptibility to CQ, desethylamodiaquine, mefloquine and lumefantrine were determined, and for CQ, isolates with an IC_50_ value > 100 nM were defined as resistant [28].

### Drug use

CQ usage within the four countries of interest in West and Central Africa was estimated using the data available from the demographic health surveys (DHS) and multiple indicator cluster surveys (MICS) [29,30]. DHS and MICS are nationally representative household surveys, which provide the data required for monitoring and assessing health indicators. They are conducted approximately every five years, using large sample sizes (between 5,000 and 30,000 households). Data on the number of febrile children under five years old that received CQ in the previous two weeks were extracted from 88 surveys in 40 African countries, using Measure DHS and United Nations Children’s Fund (UNICEF) databases between 2000 and 2011.

### Sample size calculation

In order to select eligible countries with enough data per year for significant molecular analysis, a sample size calculation, using a simple logistic regression model was used. In the model,

$$\text{logit}\left(P\right)=\beta_{0}+\beta_{1}X\text{,}$$

where P is the prevalence of mutant isolates and X is the time covariate, the null hypothesis H0: slope (β_1_) = 0 was tested for one normally distributed covariate X [31]. The sample size formula for a two-sample t-test was used:

n=Z1−α2+Z1−β2P1−Pβ*2,

with a test significance level *α* = 0.05, a power 1-*β* = 0.80, Z_u_ the upper percentile of the standard normal distribution, the event rate at the mean of X: *P* = 0.5 and β* the effect size = 0.405. The total sample size required for showing a significant increase of 10% of CQ susceptibility over time was 209 patients for each country (approximately 20 patients per year for each country).

### Statistical analyses

#### Molecular

Isolates that carried both *pfcrtK* and *pfcrtT* alleles were identified in many isolates, but the proportion of these mixed isolates proportions was constant over time, tested with a chi-square test for trend. Therefore, the prevalence of mutations at the *pfcrt76T* allele was calculated as the proportion of mutant isolates (pure + mixed genotype) out of the total of all isolates (pure mutant + wild-type + mixed genotype). With this approach, the frequency of mutant alleles in the population of isolates may be over-estimated and the frequency of wild-type allele, potentially underestimated. To describe trends in the prevalence of mutant isolates through time, a logistic regression model with a logit link function was fitted to the prevalence data with time as a linear covariate for each country. Given the probability of mutant isolates, the number of mutant isolates per year was assumed to be binomially distributed.

The estimated slopes of the logistic regression curve fitted to the observed mutant allele prevalence and the 95% confidence intervals were presented in logit scale. The slopes of the changes in prevalence among the countries were extracted from the model and compared to assess whether the slopes differed significantly from null (0) and differed significantly from each other [32].

### *In vitro*

In order to describe the temporal trends of *in vitro* data, a generalized linear model (GLM) was fitted with a log link function to IC_50_ data throughout the period 2000–2011 and after 2004:

$$\text{log}\left({\mathrm{IC}}_{50}\right)=\alpha_{0}+\alpha_{1}t\text{,}$$

where *t* is the time covariate. It was tested whether the slope *α*_*1*_ was significantly different from zero. The geometric means of the IC_50_ values per year were used to minimise the effects of outlier values. The threshold value of IC50 value > 100 nM was used as a definition of *in vitro* resistance to CQ [28].

### Drug use

The individual patient responses from each DHS or MICS survey were aggregated to give the weighted number of positive responses to CQ use. The model was fitted using a mixed effects model for 40 different African countries to account for heterogeneity across countries. The probability *P*_*ij*_ of CQ use in country *i* and survey *j* were given, as:

$$\text{logit}\left(p_{\mathrm{ij}}\right)=\alpha +\beta t_{j}+\gamma_{i}+\lambda_{i}t_{j}\text{,}$$

where *α* is the fixed effects common intercept, *β* the fixed effects common slope for the time variable *t*_*j*_ (the year of the *j*th survey), *y*_*i*_ the intercept random effect at the country level and λ_i_ the slope random effect at the country level. α + γ_i_ represents the country specific log-odds intercept for the ^*i*th^ country and *β* + *λ*_*i*_ the country specific log-odds slope of CQ use against time. By including the random effects components, the intercept and slope may differ from country to country. After fitting a model for CQ use across 40 African countries, results were extracted to estimate drug usage in Senegal, Mali, Ivory Coast and Cameroon between 2000 and 2011.

### Software

All statistical analyses were performed using Stata version 11 for Windows (Stata Corp, College Station, TX, USA) and R version 2.10.

## Results

### Travellers’ characteristics

**Figure 1:**
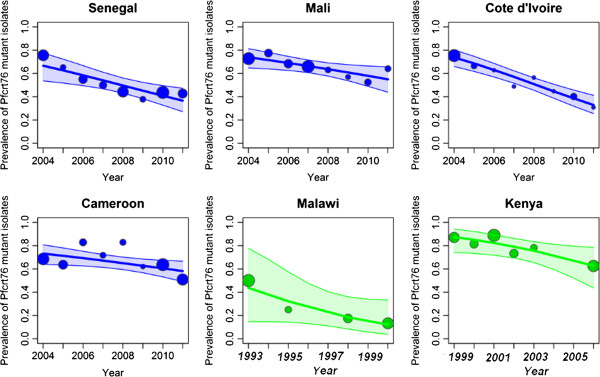
**Imported malaria infection from West and Central Africa reported in the French Malaria Surveillance system. **Number of *Plasmodium falciparum *infections diagnosed in travellers returning to France from Senegal, Mali, Ivory Coast and Cameroon notified to the National Reference Centre for Malaria (CNRpalu), Paris, France, from 2000 to 2011.

A total of 12,331 travellers infected with *P. falciparum* returned to France between 2000 to 2011 from Senegal (n = 1,970), Mali (n = 2,338), Ivory Coast (n = 4,765), and Cameroon (n = 3,258), were reported to the National Malaria Reference Centre, Paris, France (Figure 1). Of the total cases, the median age of the studied population was 31 years old, with 79% (n = 8,187) of the travellers older than 15 years old. Mainly men were infected (61%, n = 7,546). The duration of stay was more than one month for 61% (n = 6,162) of the travellers. The purpose of travel was to visit friends and relatives (VFR) for 61% (n = 6,848), living as expatriates/residents for more than six months for 14% (n = 1,572), tourism for 13% (n = 1,484), business for 5% (n = 543); and, military posting for 4% (n = 435). Only 38% (n = 4,708) reported taking prophylaxis during their travel in the endemic country. Most cases were uncomplicated malaria (95%, n = 11,146) (Table 1). The travellers returning from the four countries presented similar characteristics.

**Table 1:** Characteristics of travellers returning from Senegal, Mali, Ivory Coast, Cameroon to France, 2000–2011

**Travellers**	Senegal (n = 1,993)*	Mali (n = 2,372)*	Ivory Coast (n = 4,778 )*	Cameroon (n =3,272) *	**Total (n = 12,415)**
**Age (year) **(Median [Min-Max])	30 [0–94]	31 [0–76]	30 [0–83]	33 [0–87]	31 [0–94]
**Age class, years n **(%)					
**≤5**	78 (4)	118 (6)	349 (9)	185 (7)	730 (7)
**5-15**	247 (14)	339 (17)	585 (15)	307 (11)	1,478 (14)
**>15**	1,447 (82)	1,546 (77)	2,995 (76)	2,199 (82)	8,187 (79)
**Sex**					
Male n (%)	1,414 (71)	1,622 (69)	2,764 (58)	1,746 (54)	7,546 (61)
Female n (%)	572 (29)	736 (31)	1,990 (42)	1,512 (46)	4,810 (39)
**Chemoprophylaxis**					
No n (%)	1,113 (56)	1,182 (50)	2,485 (52)	1,993 (61)	6,773 (55)
Yes n (%)	746 (38)	959 (41)	1,955 (41)	1,048 (32)	4,708 (38)
Don’t know n (%)	124 (6)	216 (9)	322 (7)	218 (7)	880 (7)
**Duration of stay**					
≤2 weeks n (%)	218 (13)	152 (8)	457 (12)	439 (16)	1,266 (12)
2-4 weeks n (%)	356 (21)	361 (18)	1,150 (30)	868 (33)	2,735 (27)
1-3 months n (%)	699 (41)	928 (48)	1,221 (32)	679 (25)	3,527 (35)
>3 months n (%)	428 (25)	498 (26)	1,021 (26)	688 (26)	2,635 (26)
**Purpose of travel**					
VFRs n (%)	1,108 (61)	1,520 (71)	2,482 (58)	1,738 (59)	6,848 (61)
Residents or expatriates ≥6 months n (%)	237 (13)	207 (9)	568 (13)	560 (19)	1,572 (14)
Tourism n (%)	322 (18)	251 (12)	514 (12)	397 (13)	1,484 (13)
Business n (%)	71 (4)	90 (4)	212 (5)	170 (6)	543 (5)
Military n (%)	24 (1)	15 (1)	368 (9)	28 (1)	435 (4)
Other n (%)	48 (3)	67 (3)	142 (3)	68 (2)	325 (3)
**Severe malaria**					
No n (%)	1,834 (93)	2,235 (95)	4,513 (95)	3,064 (95)	11,146 (95)
Yes n (%)	136 (7)	123 (5)	225 (5)	177 (5)	661 (5)
**No *pfcrt76 *analyses**	594	701	860	719	2874
**No *In vitro *analyses for CQ**	305	396	729	513	1483

*Numbers may not add to totals because of missing values.

### Molecular results

The overall goal was to compare the trends in resistance, parasite susceptibility to CQ and the relationship of these values to an estimate of actual drug use in each country. From 2000 to 2011, the prevalence of the *pfcrt76T* allele in parasites from patients returning from Senegal significantly decreased from 78% to 47% (slope = −0.17, p < 10^-3^); from 63% to 37% for Ivory Coast (slope = −0.15, p < 10^-3^); and, from 90% to 59% for Cameroon (slope = −0.09, p < 10^-3^) but no significant decrease of CQ-resistant isolates was observed for Mali (slope = −0.01, p = 0.72) (Table 2).

**Table 2:** **Trends in molecular genotypes and *in vitro *susceptibility for *P. falciparum *isolates**

**Slopes of prevalence in *Pfcrt76T***				
	**2000-2011**		**2004-2011**	
	**Slope (95% CI)**	**P value**	**Slope (95% CI)**	**P value**
Senegal	−0.167 [−0.209; -0.125]	<10^-3^	−0.182 [−0.264; -0.102]	<10^-3^
Mali	−0.009 [−0.052; 0.034]	0.72	−0.102 [−0.178; -0.027]	0.03
Ivory Coast	−0.146 [−0.183; -0.111]	<10^-3^	−0.265 [−0.325; -0.207]	<10^-3^
Cameroon	−0.090 [−0.130; -0.050]	<10^-3^	−0.106 [−0.172; -0.041]	<10^-3^
**Slopes of IC**_**50 **_**values for CQ**				
Senegal	−0.081 [−0.130; -0.032]	<10^-3^	−0.123 [−0.220; -0.026]	<10^-3^
Mali	−0.042 [−0.090; 0.007]	0.02	−0.098 [−0.167; -0.030]	<10^-3^
Ivory Coast	−0.057 [−0.093; -0.021]	<10^-3^	−0.120 [−0.177; -0.063]	<10^-3^
Cameroon	−0.055 [−0.089; -0.020]	<10^-3^	−0.100 [−0.154; -0.046]	<10^-3^

Slopes of prevalence in *Pfcrt76T *and slopes of IC50 values from patients returning to France from Senegal, Mali, Ivory Coast, and Cameroon, between 2000–2011 and 2004–2011 (after CQ withdrawal).

**Figure 2:**
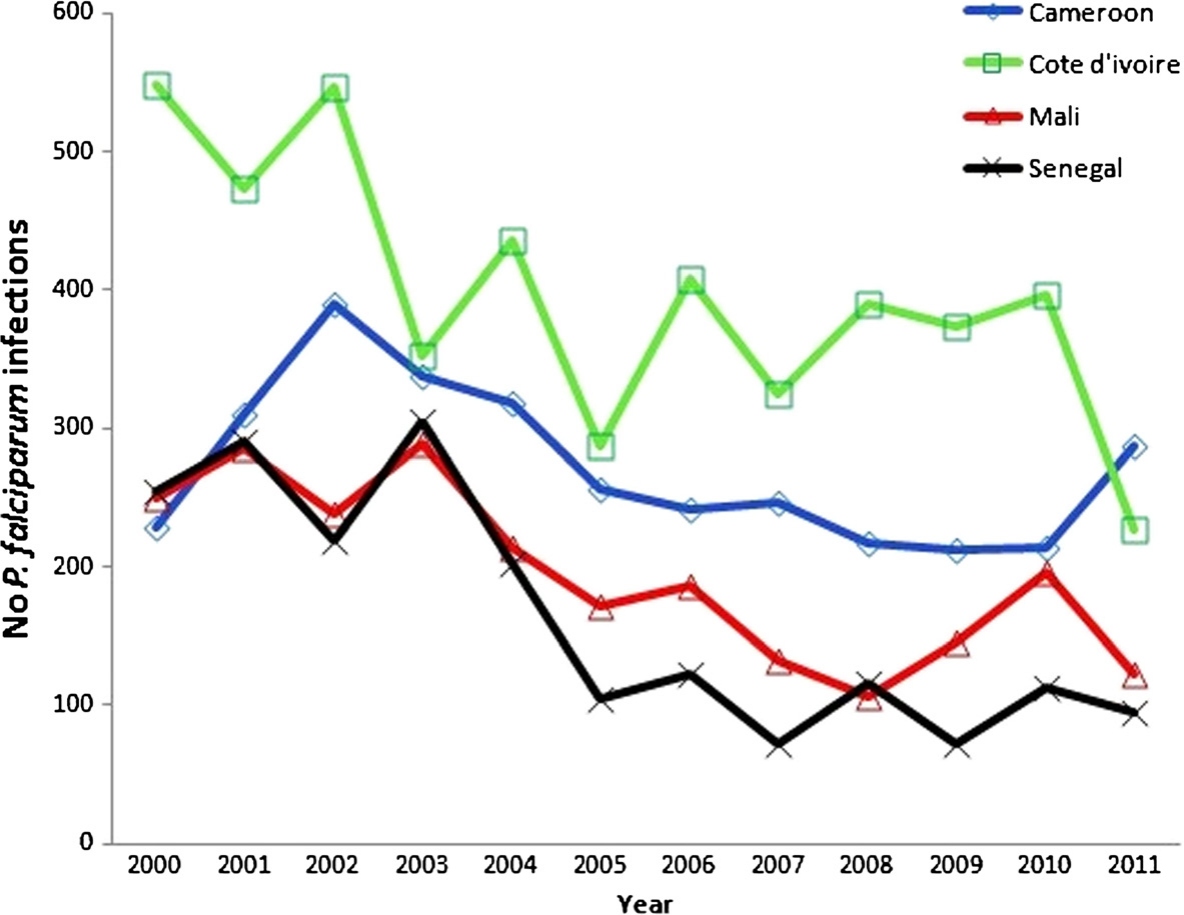
**Evolution of the prevalence of *pfcrt76T *isolates after the CQ ban in different African regions. **Observed and fitted (by logistic regression) prevalence of *pfcrt76 *mutant isolates and 95% Confidence Interval, from travellers returning to France from **A**) Senegal, **B**) Mali **C**) Ivory Coast, and **D**) Cameroon, from 2004 to 2011, after that most African countries officially banned the use of CQ. The slopes and their standard error are displayed for each country. Each spot represents the number of isolates per year for travellers’ data and per study for field studies. The size of the spot is proportional to the number of isolates. The p-value indicates whether the slope of the predicted line is significantly different from zero (data from CNRpalu, France). These data are compared with two field studies **E**) in Malawi and **F**) Kenya after the CQ ban (1993 for Malawi and 1999 for Kenya) [14,16].

By 2004, CQ use had been discontinued in all of these countries. The same analyses were repeated over the period 2004–2011. The decrease in the prevalence of the *pfcrt76T* genotype was significant and faster in all cases than during the period 2000–2011. In Senegal (slope = −0.18, p < 10^-3^), Mali (slope = −0.10, p = 0.03), Ivory Coast (slope = −0.27, p < 10^-3^) and Cameroon (slope = −0.11, p < 10^-3^) the prevalence decreased (Figure 2). When the mixed genotype isolates were removed from the analyses, the same trends were observed. To compare these trends with those from other regions, published data sets of similar molecular data from Kenya and Malawi, after CQ withdrawal, were accessed and the logistic regression model fitted to these data [14,16]. The slopes of the prevalence of *pfcrt76T* were strongly negative: Malawi, 1993–2000 (slope = −0.25, p < 10^-3^) and Kenya, 1999–2006 (slope = −0.20, p = 0.003) (Figure 2). However, comparison of the slopes showed no significant differences among the six countries (p = 0.22).

### *In vitro* results

A second way of assessing changes in CQ susceptibility was to measure the response of isolates *in vitro*. From 2000 to 2011, the geometric mean of the IC_50_ of CQ of the isolates tested *in vitro* by the ^3^H-hypoxanthine uptake inhibition method decreased significantly in each country. In Senegal, the value decreased from 86 nM (95% confidence interval [95% CI], 51–145, 59% resistant) to 38 nM (95% CI, 25–59, 25% resistant); from 84 nM (95% CI, 35–198, 50% resistant) to 51 nM (95% CI, 36–73, 31% CQ resistant) in Mali; from 75 nM (95% CI, 43–130, 59% resistant) to 30 nM (95% CI, 22–40, 16% resistant) in Ivory Coast and from 181 nM (95% CI, 87–374, 75% resistant) to 50 nM (95% CI, 36–69, 37% resistant) in Cameroon (Table 2).

**Figure 3:**
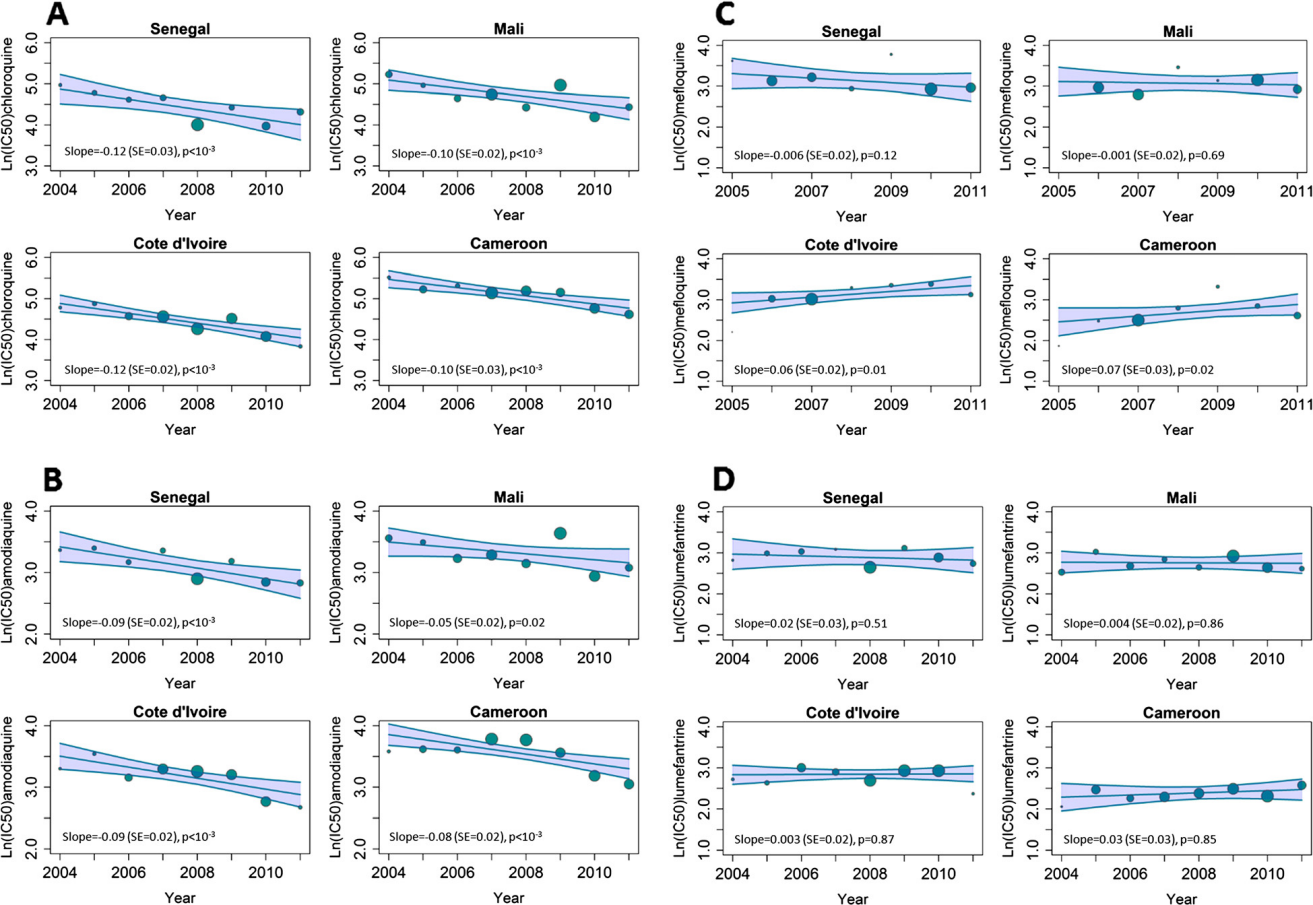
**Evolution of *in vitro *anti-malarial drug response in imported malaria. **A generalized linear model is fitted to the *in vitro *data **A**: chloroquine response (IC_50_), **B**: desethylamodiaquine response, **C**: mefloquine response, **D**: lumefantrine response, for clinical isolates collected from travellers returning to France from Senegal, Mali, Ivory Coast, and Cameroon, from 2004 to 2011. Slopes, standard errors and the p-value indicating whether the slope of the predicted line is significantly different from zero are displayed (data from CNRpalu, France). Each spot represents the geometric mean IC_50 _per year and the size of the spot is proportional to the number of isolates.

Constraining the analyses over the period 2004–2011 resulted in an even faster decrease of *in vitro* susceptibility for CQ for the four African countries: Cameroon (slope = −0.10, p < 10^-3^), Ivory Coast (slope = −0.12, p < 10^-3^), Mali (slope = −0.10, p < 10^-3^) and Senegal (slope = −0.12, p < 10^-3^) (Figure 3A, Table 2).

Susceptibility to other anti-malarial drugs, i.e., amodiaquine, mefloquine and lumefantrine was also examined as they are components of artemisinin combination therapy currently in use. Moreover, some *in vitro* studies have suggested that there is cross-resistance between amodiaquine and CQ because of their similar chemical structure [33]. Mefloquine and lumefantrine belong to the amino-alcohol class and some *in vitro* studies have also suggested an inverse relationship between the responses of CQ and amino-alcohols [34,35]. The *in vitro* responses of isolates for these other drugs were also determined for the period 2004–2011. The geometric mean of the IC_50_ values for desethylamodiaquine (active metabolite of amodiaquine) showed a significant decline in each of the four countries (Figure 3B). Susceptibility to mefloquine showed an increasing trend in Ivory Coast and Cameroon, but the values were stable in Senegal and Mali (Figure 3C). In contrast, the susceptibility *in vitro* to lumefantrine was stable in all four countries (Figure 3D).

The *in vitro* and molecular methods are indirect indicators of parasite resistance to anti-malarial drugs, but allow efficient longitudinal and temporal surveillance of any changes. In particular, these approaches have been used to track the trends in resistance following the change from CQ to other drugs as recommended first line therapies [14,16,36]. The actual drug use in a country is, of course, a key parameter in such changes. To examine these trends, the drug use data were extracted from DHS and MICS surveys in the four target countries, applying a mixed effects model. The percentage usage of CQ in children with fever in 88 surveys from 40 African countries showed the general decrease in use from 2000 to 2011 (fixed effects slope = −0.3, p <10^-3^; random effects slope = 0.076, p <10^-3^). The significant decrease during this period was more specifically observed in Senegal, from 38% to 2% (slope = −0.33, p < 0.01); from 42% to 13% in Mali (slope = −0.16, p < 0.01); from 56% to 18% in Ivory Coast (slope = −0.18, p < 0.01); and from 49% to 2% in Cameroon (slope = −0.37, p < 0.01), after extracting results from the mixed effects model.

**Figure 4:**
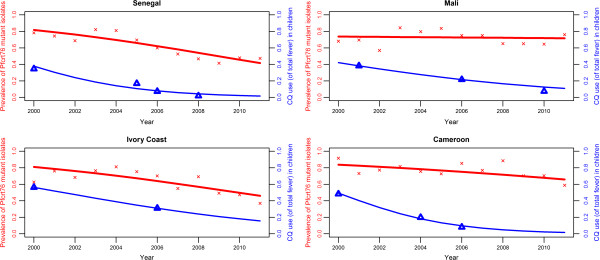
**Evolution of the prevalence of *pfcrt76 *mutant isolates regarding the change in drug use, 2000–2011. **The Y-axis represents the observed (red crosses) and fitted (by logistic regression, red line) prevalence of *pfcrt76 *mutant isolates from travellers returning to France from Senegal, Mali, Ivory Coast and Cameroon, from 2000 to 2011 (data from CNRpalu, France). The Z-axis represents observed (blue upward triangle) and fitted (by logistic regression using linear mixed model, blue line) prevalence of CQ use among fever in children under five years-old with fever in Senegal, Mali, Ivory Coast and Cameroon, from 2000 to 2011 (data from Demographic Health Survey and Multiple Indicator Cluster Survey).

Using these estimates of trends in CQ use, there was a positive correlation between CQ use and prevalence of the *pfcrt76T* allele in Senegal (r^2^ = 0.79, p < 0.01) and Ivory Coast (r^2^ = 0.74, p < 0.01) but not in Mali (r^2^ = 0.18, p = 0.57) or Cameroon (r^2^ = 0.45, p = 0.14) (Figure 4).

## Discussion

The present study suggested a progressive return of CQ susceptibility in four countries of West and Central Africa, i.e., Senegal, Mali, Ivory Coast and Cameroon, based on the surveillance of patients returning to non-endemic areas with *P. falciparum* infection from 2000–2011. These results, from both molecular and *in vitro* analyses, show that CQ resistance was in decline during this period, except in Mali. The same analyses performed for the period 2004–2011, after these countries had changed their national recommendations from CQ to alternative anti-malarial drugs, confirm the increase of CQ susceptibility and show an accentuated trend compared to the period 2000–2011, in Senegal, Ivory Coast and Cameroon. A significant decrease of CQ resistance was observed in Mali only after 2004.

The conclusions from these indirect measures of CQ efficacy in travellers are consistent with similar measures assessed in the field. For Senegal, the prevalence of *pfcrt76T* isolates in travellers showed a similar trend as observed in two studies conducted in Dakar, but a different trend from a study conducted in the city of Pikine, located 15 km from Dakar [18,19,37]. These results are also consistent with the East African countries, Malawi and Kenya, where the return of CQ susceptibility was observed following the official withdrawal of this drug. Indeed, the prevalence of *pfcrt76T* mutant isolates in travellers is comparable with previously published data from the field in East Africa after the CQ ban and similar trends are described (Figure 2).

Although the trends are similar, there are variations among the countries in the trends and many factors are likely to explain these. Overall, the main factor is the efficiency with which new drug policies are implemented. This is, in turn, strongly influenced by the political, economic, geographic, social and cultural contexts within each country and region. The pharmaceutical distribution modes and drug supply chains impact the use of drugs, depending on the level of control that the gover nMent exercises over the pharmaceutical distribution system in the public and private sectors [38,39]. For example, Malawi, which implemented successful national information campaigns and efficient control of its distribution, was able to almost completely ban CQ use and show, after 10 years, a complete recovery of the CQ susceptibility [40]. In addition, overall malaria prevalence the distance between patients and public health facilities, and socio-economic level, age of patients and have also been identified as key factors for CQ use [41,42]. In the West African region, migration of people carrying parasites displaced from other regions is also a potentially important factor. Deeper analysis in countries that differ greatly in the response to withdrawal of a drug will help to quantify the influence of these factors.

Despite the disparate influences, the overall effectiveness of drug policy implementation has important and far-reaching effects on the useful therapeutic life of anti-malarial drugs by delaying the emergence of resistance.

Most important, following the emergence of resistance, the time between official policy changes and their subsequent implementation, directly impacts public health; use of poorly effective drugs increases malaria mortality and morbidity [10,11]. This consequence has been confirmed in some studies which reported the decrease in the proportion of severe malaria cases and in malaria-specific mortality after the introduction or distribution of free ACT [43-45].

The trends of CQ use, which were estimated using DHS and MICS data, show a steady decrease for the four countries of interest without major differences among them. However, the slow decrease in the prevalence of the *pfcrt76T* allele observed in Mali might be partly explained by the relatively slow decrease of CQ consumption illustrated in Figure 4. The reversal from high prevalence of the resistant mutant genotype *pfcrt76T* within the parasite population to the wild type genotype *pfcrtK76* might be explained by the fitness cost of the resistant mutant: there is evidence that the parasites that carry the wild type *pfcrt76K* allele have a survival advantage in the absence of drug pressure [40]. However, the positive correlation between CQ use and prevalence of CQ resistance was only shown for Senegal and Ivory Coast. Beyond country-specific drug policies and efficacies in implementing them, other factors may also play a role in differences between countries. For example, transmission intensity, which differs between sites, might impact the CQ use and therefore, the prevalence of mutant isolates. This is observed with reversion back to the 76 K haplotype occurring during low transmission seasons [46,47]. Multiple first-line therapies or cycling strategies, where anti-malarial therapies are rotated, might be one of the tools to decrease drug pressure and help prevent the spread of resistance [48].

The four West and Central African countries currently recommend the combination of artesunate-amodiaquine as first-line treatment [49]. In addition to the recommended first-line drugs, many other anti-malarials are commonly used in these countries and selection pressures on the *pfcrt* locus are complex. In Southeast Asia, addition of artesunate to mefloquine was followed by a partial reversal of mefloquine resistance [50,51].

Amodiaquine is closely related to CQ, and was also used considerably in these four countries. The change from monotherapy with amodiaquine in combination with artesunate may have provided reciprocal protection to the artemisinin derivative and to the partner drug influenced the return of amodiaquine and CQ susceptibility. It is, therefore, possible that the significant decrease of IC_50_ for desethylamodiaquine might be explained both by the decrease of CQ-resistant isolates and the switch from amodiaquine monotherapy to artesunate-amodiaquine combination therapy in West and East African region after 2006. The decrease of *in vitro* mefloquine susceptibility in Cameroon and Ivory Coast may have selected CQ-susceptible isolates. Indeed, the inverse correlation between CQ and mefloquine, which was described in previous studies, could explain the opposite trend [35,52].

The present study has several limitations worth noting. Returning travellers are not a representative sample of the native population and the precise location within the country where infection occurred is not reported. However, trends for *in vitro* and molecular results for imported malaria are similar to those observed in previous publications from Senegal [18,19]. For validation of this approach, these results should be compared to country-level *in vitro* and molecular data analysed in the same conditions for the four countries. There are also some limitations and bias regarding the consumption data. The CQ use in children under five years old with fever is used as a proxy of the CQ consumption in the country. The data are only based on the mother’s declaration and do not assess directly the blood drug concentration. This approach is less reliable than biological methods because of misunderstanding of questions, failed memory or deliberate attempts to provide false information [53].

Emergence of resistance to artemisinin has also been confirmed recently in different loci in Southeast Asia [54,55]. This raises concerns about the potential spread of this resistance in sub-Saharan Africa, as has been previously described for other anti-malarial drugs. Effective surveillance systems to monitor anti-malarial drug resistance in Africa are essential to protect the efficacy of ACT. So, despite the limitations, surveillance of parasites from travellers can be used to monitor the evolution of resistance over time, and can provide useful information, especially from areas where little information is available. Those data can be realised rapidly and methods can be more easily standardized. This non-immune population, unlikely to be re-infected, also facilitates detection of resistant isolates and true clinical failure.

## Conclusions

The longitudinal pattern of CQ resistance in four West and Central African countries using travellers’ data was described and the CQ withdrawal after policy change may have accelerated the return of CQ susceptibility. The length of time between policy changes and their subsequent implementation, as well as the use of analogue anti-malarial drugs, may affect the time for a significant recovery of CQ sensitivity. Despite the regain of CQ susceptibility, any reintroduction would likely to result in a rapid re-emergence of resistance strains [56]. This study highlights the correlation between drug pressure and resistance prevalence [57]. The rapidly evolving pattern of anti-malarial drug resistance in endemic countries emphasises the need for a sustainable surveillance system, which would enable the implementation of more longitudinal studies.

## Competing interests

The authors declare that they have no competing interests.

## Authors’ contributions

MG, PJG and JL conceived and designed the study and interpreted the results; MG conducted the research and wrote the manuscript; MG and JAF performed the statistical analysis; VH and LB carried out the biological analysis; EK was responsible for the travellers’ surveillance database; JEM participated in the collection of drug use data; PJG and JL supervised the manuscript; the CNR study group participated in samples and data collection. All authors read and approved the final manuscript.

## Authors’ information

Members of the French National Reference Center for Imported Malaria Study.

Ahmed Aboubacar, Patrice Agnamey, Adela Angoulvant, Patricia Barbut, Didier Basset, Ghania Belkadi, Anne Pauline Bellanger, Dieudonné Bemba, Françoise Benoit-Vical, Antoine Berry, Marie-Laure Bigel, Julie Bonhomme, Françoise Botterel, Olivier Bouchaud, Marie-Elisabeth Bougnoux, Patrice Bourée, Nathalie Bourgeois, Catherine Branger, Laurent Bret, Bernadette Buret, Enrique Casalino, Sylviane Chevrier, Frédérique Conquere de Monbrison, Bernadette Cuisenier, Martin Danis, Marie-Laure Darde, Ludovic De Gentile, Jean-Marie Delarbre, Pascal Delaunay, Anne Delaval, Guillaume Desoubeaux, Michel Develoux, Jean Dunand, Rémy Durand, Odile Eloy, Nathalie Fauchet, Bernard Faugere, Albert Faye, Odile Fenneteau, Pierre Flori, Madeleine Fontrouge, Chantal Garabedian, Françoise Gayandrieu, Nadine Godineau, Pascal Houzé, Sandrine Houzé, Jean-Pierre Hurst, Houria Ichou, Laurence Lachaud, Agathe Lebuisson, Magalie Lefevre, Anne-Sophie Le Guern, Gwenaël Le Moal, Daniel Lusina, Marie-Claude Machouart, Denis Malvy, Sophie Matheron, Danièle Maubon, Denis Mechali, Bruno Megarbane, Guillaume Menard, Laurence Millon, Muriel Mimoun Aiach, Philippe Minodier, Christelle Morelle, Gilles Nevez, Philippe Parola, Daniel Parzy, Olivier Patey, Pierre Patoz, Pascale Penn, Alice Perignon, Stéphane Picot, Jean-Etienne Pilo, Isabelle Poilane, Denis Pons, Marie Poupart, Bruno Pradines, Didier Raffenot, Christophe Rapp, Marie-Catherine Receveur, Claudine Sarfati, Yaye Senghor, Fabrice Simon, Jean-Yves Siriez, Nicolas Taudon, Marc Thellier, Maxime Thouvenin, Dominique Toubas.
